# Impact of Ultrasound-Guided Deep Serratus Anterior Plane Block Combined With Dexmedetomidine as an Adjuvant to Ropivacaine Inpatient Quality of Recovery Scores Undergoing Modified Radical Mastectomy: A Randomized Controlled Trial

**DOI:** 10.3389/fonc.2022.858030

**Published:** 2022-03-31

**Authors:** Yu Wu, Yuling Kang, Yanli Li, Bohan Fu

**Affiliations:** Department of Anesthesiology, Bethune International Peace Hospital, Shijiazhuang, China

**Keywords:** modified radical mastectomy, serratus anterior plane block, dexmedetomidine, QoR-15, enhanced recovery after surgery

## Abstract

**Background:**

Breast cancer has overtaken lung cancer as the most commonly diagnosed malignancy and is the leading cause of cancer-related death in women. Surgery is the only possible cure for breast cancer, and the incidence of acute postoperative pain (APP) is high in breast surgery. Previous reports suggested that ultrasound-guided deep serratus anterior plane block (dSAPB) provided effective blockade to relieve pain after modified radical mastectomy for breast cancer. In fact, despite the long-acting local anesthetic agents used, the patient’s pain cannot completely be eliminated due to the short duration of anesthesia. Dexmedetomidine as an adjunct to local anesthetics can prolong peripheral nerve block duration. However, no study has investigated the role of dSAPB with dexmedetomidine in the quality of recovery scores undergoing modified radical mastectomy. Thus, this study was conducted aiming at this aspect.

**Material and Methods:**

This single-center, double-blind, randomized clinical trial was conducted at Bethune International Peace Hospital. A total of 88 participants of elective modified radical mastectomy were enrolled from May and November 2021. Ultrasound-guided dSAPB combined with 30 ml of 0.375% ropivacaine or 30 ml of 0.375% ropivacaine with dexmedetomidine (1 μg/kg) was administrated before anesthesia at the fourth to fifth ribs of the axillary midline. The primary outcome was quality of recovery, measured 24 h postoperatively using the QoR-15. Secondary outcomes were the Visual Analogue Scale (VAS) scores at rest and movement at 1, 6, 12, 24, and 48 h after surgery, 48 h sufentanil consumption postoperatively, the incidence of postoperative nausea and vomiting (PONV), length of post-anesthesia care unit (PACU) stay, dizziness, delirium, SAPB-related adverse events, and patient’s satisfaction with pain management.

**Results:**

Among the 88 participants, 8 did not meet the inclusion criteria; the other 80 were randomized to receive dSAPB combined with ropivacaine (Group R, N=40) and dSAPB combined with ropivacaine plus DEX (Group RD, N=40), of which a total of 7 (4 in Group R and 3 in Group RD) were excluded due to protocol deviation. Eventually,73 participants (36 in Group R and 37 in Group RD) were included for final analysis, with age (SD, years, 54.08[6.28] vs. 54.62[7.44], p=0.740), body mass index (BMI) (SD, 27.96[1.67] vs. 27.57[2.38], p=0.428), and median preoperative global QoR-15 score (interquartile range (IQR), 127[123.25–131] vs. 126[121–130], p=0.662). The median postoperative global QoR-15 score (IQR, 107[103–112] vs. 109.5[107–114], p=0.016), VAS score at rest at 12th hour (IQR, 1[1–2] vs. 1[1–2], p=0.033), VAS score in movement at 12th hour (IQR, 2[1–3] vs. 2[1–3], p=0.014) and at 24th hour (IQR, 3[2–3] vs. 3[2–3], p=0.040), and median sufentanil rescues consumption (IQR, 14[12–17 vs. 14[12–15], p=0.022] of Group RD were significantly lower than those of the Group R. Patient satisfaction score (SD, 8.28[0.70] vs. 8.62[0.59], p=0.024) of Group RD were significantly higher than those of the Group R.

**Conclusion:**

The ultrasound-guided dSAPB combined with dexmedetomidine plus ropivacaine may improve the QoR-15 in patients undergoing modified radical mastectomy and indicates that it may be a useful intervention to aid recovery following breast cancer surgery. Furthermore, participants in the ropivacaine with DEX group met the superior pain relief in the early postoperative period, reduced postoperative cumulative opioid consumption, increased patient satisfaction, and no increase in the incidence of complications.

## Introduction

Breast cancer has overtaken lung cancer as the most commonly diagnosed malignancy and is the leading cause of cancer-related death in women (1, 2). During the past recent years, various therapies emerged in the era of breast cancer. Breast cancer is a heterogeneous disease in which genetic and environmental factors are involved (3). Despite that the breast cancer age-standardized mortality rates have decreased by 2%–4% per year since the 1990s (4), the incidence of breast cancer was 11.7% in total new cases in 2020, both sexes and in all ages (5). Breast cancer treatment methods include surgery, radiotherapy, chemotherapy, hormone therapy, targeted therapy, and immunotherapy (6). Surgery is the only possible cure for breast cancer, so surgical resection of the tumor is the preferred treatment for early breast cancer (7, 8).

The incidence of acute postoperative pain (APP) is high in breast surgery, and opioids are the most commonly used drugs to treat APP. However, they are not without systemic side effects, which may increase comorbidities (9). Although there is no evidence that perioperative pain management reduces patient mortality, perioperative pain might influence the oncological outcome in major tumor resection surgery (10, 11).

There are some local or regional nerve blocks in breast cancer performed as core components of multimodal analgesia and enhanced recovery after surgery (ERAS) (12), including thoracic epidural (13), interscalene brachial plexus (14), paravertebral (15), pectoral nerve blocks (16, 17), and erector spinae plane block (18). Ultrasound-guided serratus anterior plane block (SAPB) is a new analgesic technique applied to the clinic proposed by Blanco (19); it is a block in which local anesthetic is deposited within an interfascial plane either superficial or deep into the serratus anterior muscle at the mid-axillary line (20). Some clinical trials reported that both superficial and deep SAPB provided effective blockade to alleviate the pain after modified radical mastectomy for breast cancer (21–23), but Edwards et al. suggested that the deep SAPB may improve analgesia to a greater degree than the superficial SAPB (24). In fact, despite the use of long-acting local anesthetic agents, the patient’s pain cannot completely be eliminated due to the short duration of anesthesia. Dexmedetomidine is a highly selective alpha-2 adrenal receptor agonist (25); as an adjunct to local anesthetics, the duration of peripheral nerve block can be prolonged. Notably, dexmedetomidine assisted with local anesthetics has been reported to accelerate the onset of action and prolong the duration of block (18, 26, 27). However, to the best of our knowledge, there are no reports on the efficacy and safety of dexmedetomidine combined with local anesthetic in dSAPB. The purpose of this study was to compare the efficacy of dexmedetomidine combined with dSAPB in breast cancer surgery.

## Methods

### Study Participants

The study was a prospective, single-center, parallel-group, randomized, double-blind clinical trial, approved by the ethics committee of Bethune International Peace Hospital, Shijiazhuang, Hebei Province, China (2021-KY-128), and registered in the Chinese Clinical Trial Registry (ChiCTR2100045264). This study followed the Consolidated Standards of Reporting Trials (CONSORT) statement and principles of the Declaration of Helsinki. One day before surgery, all patients were evaluated and signed written informed consent to participate in the trial. A total of 88 female patients aged 33–65 years with BMI ≤30 kg/m^2^ and American Society of Anesthesiologists (ASA) I–II who were scheduled for modified radical mastectomy for breast cancer between May and November 2021 were included in this study. The exclusion criteria included a history of allergy to any trial drugs, presence of coagulation disorders, infection at the nerve block puncture site, ingestion of any analgesic drug within 48 h before surgery, history of chronic pain medication use, trauma or history of thoracic spine surgery, sinus bradycardia, and atrioventricular block and any other conditions that were not appropriate for this study.

### Random Selection of Patients

The study participants were using a computer-generated list of random numbers, randomly grouped on a scale of 1:1. The distribution results were sealed in an opaque envelope and kept by the study manager. On the surgical day, the study administrator handed the envelope to the anesthesia assistant who dispensed the anesthetic fluid. The patients were randomly assigned to two groups: the ropivacaine group (Group R) and the ropivacaine plus dexmedetomidine group (Group RD), with 36 patients in each group. An indwelling intravenous needle and noninvasive blood pressure, electrocardiogram, oxygen saturation, and Bispectral index were monitored to the patient who entered the operating room. Before the nerve block, all patients received intravenous 1 μg/kg of dexmedetomidine for 10 min, and 5 ml of 0.9% isotonic saline was prepared for positioning. The anesthesia assistant prepared a total of 30 ml of 0.375% ropivacaine or 30 ml of 0.375% ropivacaine with dexmedetomidine (1 μg/kg). The assistant handed the syringe filled with the potion to the anesthesiologist, who has mastered the anesthesia but did not know which group the patient belongs to. Thus, group assignments were blinded by patients, healthcare providers (anesthesiologists who performed SAPB and were responsible for intraoperative care and surgeons), and data collectors.

### Deep Serratus Anterior Plane Block Process

After patients entered the operating room and received intravenous dexmedetomidine of 1 μg/kg for 10 min, the patient was placed in the lateral decubitus position, facing upward, with the patient’s independent arm comfortably resting above the head, exposing the lateral chest wall. A 6–13-MHz linear array ultrasonic sensor probe (HFL38x; FUJIFILM SonoSite, Bothell, Washington) identified the fourth to fifth ribs of the axillary midline. Intercostal muscles between the ribs and the serratus anterior muscles and the latissimus dorsi muscles on the surface of the ribs were identified. The thoracic dorsal artery is also identified as an additional anatomical marker to identify the serratus anterior superficial plane. The skin at the needle insertion site was sterilized with 1% iodophor, and 1% lidocaine was used for local anesthesia where block needles enter into the skin. Under the guidance of continuous ultrasound, a short inclined non-stimulating puncture needle (Pajunk, 22 gauge) was inserted through the skin wound, and the needle was pushed with saline until the puncture tip was on the appropriate plane; then, ropivacaine with or without dexmedetomidine was deposited and adequately spread in schedule, respectively, and confirmed by direct ultrasound visualization.

### Standard Procedure for General Anesthesia

Standardized general anesthesia regimens for all patients. After a 3-min preoxygenation, general anesthesia was induced with 0.1 mg/kg of dezocine, 2 mg/kg of etomidate, 2 mg/kg of propofol, and 0.4 μg/kg of sufentanil intravenously after the nerve block was completed. Upon loss of consciousness, 0.6 mg/kg of rocuronium was used for laryngeal mask airway (LMA) placement. All patients were given 10 mg of dexamethasone and 5 mg of tropisetron immediately after induction of anesthesia. To maintain anesthesia in the patients, 4–6 mg/kg of propofol and 0.1–1 μg/kg of remifentanil were used; 0.2 mg/kg rocuronium was maintained for 40–60 min intraoperatively. Sufentanil (0.1 μg/kg) was intravenously injected when the patient’s hemodynamic parameters exceeded 20% of the baseline. Patients were given 0.25 mg of atropine at a heart rate below 50 beats/min. The bispectral index system value during anesthesia maintenance was between 40 and 60. Pressure-controlled ventilation was used to maintain end-tide partial pressure of carbon dioxide (PaCO_2_) between 35 and 45 mmHg. At the end of the surgery, if necessary, 1 mg of neostigmine and 0.5 mg of atropine were antagonized with neuromuscular blockers. After the LMA was removed, the patients were transferred to the post-anesthesia care unit (PACU) for observation. The postoperative analgesic regimen consisted of routine intravenous administration of 50 mg of flurbiprofen every 12 h and patient-controlled intravenous analgesia (PCIA) accompanied by sufentanil (PCIA), which was set to deliver a bolus dose of 4 μg sufentanil (2 μg/ml) on-demand, with a lockout interval of 15 min and without basal infusion. Postoperative pain was assessed using a Visual Analogue Scale (VAS) on a scale of 0 (no pain) to 10 (the worst pain imaginable). If the VAS pain score exceeded 3 or the patient required, intravenous 2 μg of sufentanil was administered as a rescue analgesic by using the PCIA device. Five milligrams of tropisetron and 10 mg of metoclopramide were administered intravenously, if postoperative nausea or vomiting occurred, as rescue antiemetics.

### Primary and Secondary Outcomes

This study was conducted to determine whether preoperative deep serratus anterior plane block combined with ropivacaine or ropivacaine plus dexmedetomidine is superior in improving the quality of rehabilitation after modified radical mastectomy. The primary outcome was quality of recovery, measured 24 h postoperatively using the QoR-15, a development and Psychometric Evaluation of a Postoperative Quality of Recovery Score (28, 29), which comprises five domains of testing: pain (two questions), physical comfort (five questions), physical independence (two questions), psychological support (two questions), and emotional state (four questions).

Secondary outcomes were the VAS scores at rest and movement at 1, 6, 12, 24, and 48 h after surgery, 48 h sufentanil consumption postoperatively, the incidence of postoperative nausea and vomiting (PONV), length of PACU stay, dizziness, delirium, SAPB-related adverse events, and patient’s satisfaction with pain management. The interval as PACU admission to the Aldrete score reaching 9 was defined as the length of PACU stays (30). Patient satisfaction with pain management was assessed 24 h after surgery using an 11-point Likert scale (range, 0–10; 0 equals entirely unsatisfied, and 10 equals fully satisfied) (31). All the above results were assessed by an independent researcher unaware of the group assignment.

### Sample Size Estimation

The sample size was calculated based on global QoR-15 points 24 h after surgery. A change of 8.0 on the QoR-15 score was considered as a clinically significant improvement in QoR after surgery and anesthesia (32). According to the pilot study, the QoR-15 scores at 24 h postoperatively were equivalent to 117.6 (11.2) in the dSAPB with the ropivacaine group. Assuming a two-tailed alpha threshold of 0.05 and a power (1-beta) of 90% to detect an increment of 8.0 in the QoR-15 scores at a significance, 36 participants in each group were required. Taking into consideration a 20% withdrawal and loss for follow-up, we finally recruited 88 patients in this study.

### Statistical Analysis

All statistical analyses were performed using IBM SPSS software Version.23.0 (IBM, Armonk, NY: IBM Corp). The normality of quantitative variables was examined with the Kolmogorov–Smirnov test. Quantitative variables are expressed as mean or median (IQR). Student’s t-test was used to compare the mean values of age, weight, height, operation time, and PACU discharge time. The overall QoR-15 score and the cumulative use of sufentanil after surgery are reported as the median (interquartile range [IQR]) and were analyzed using the Mann–Whitney U-test. A 95% CI of differences was given for each statistical comparison. Categorical variables were reported as numbers and percentages. The proportion of ASA classification and the number of PONV patients were compared by χ^2^-test. Fisher’s precise test was used to compare rates of dizziness or delirium between groups. In addition, analysis of variance was performed on multiple comparisons to assess pain scores within 24 h after surgery. A p-value <0.05 was considered statistically significant in the two-sided test.

## Results

**Figure 1:**
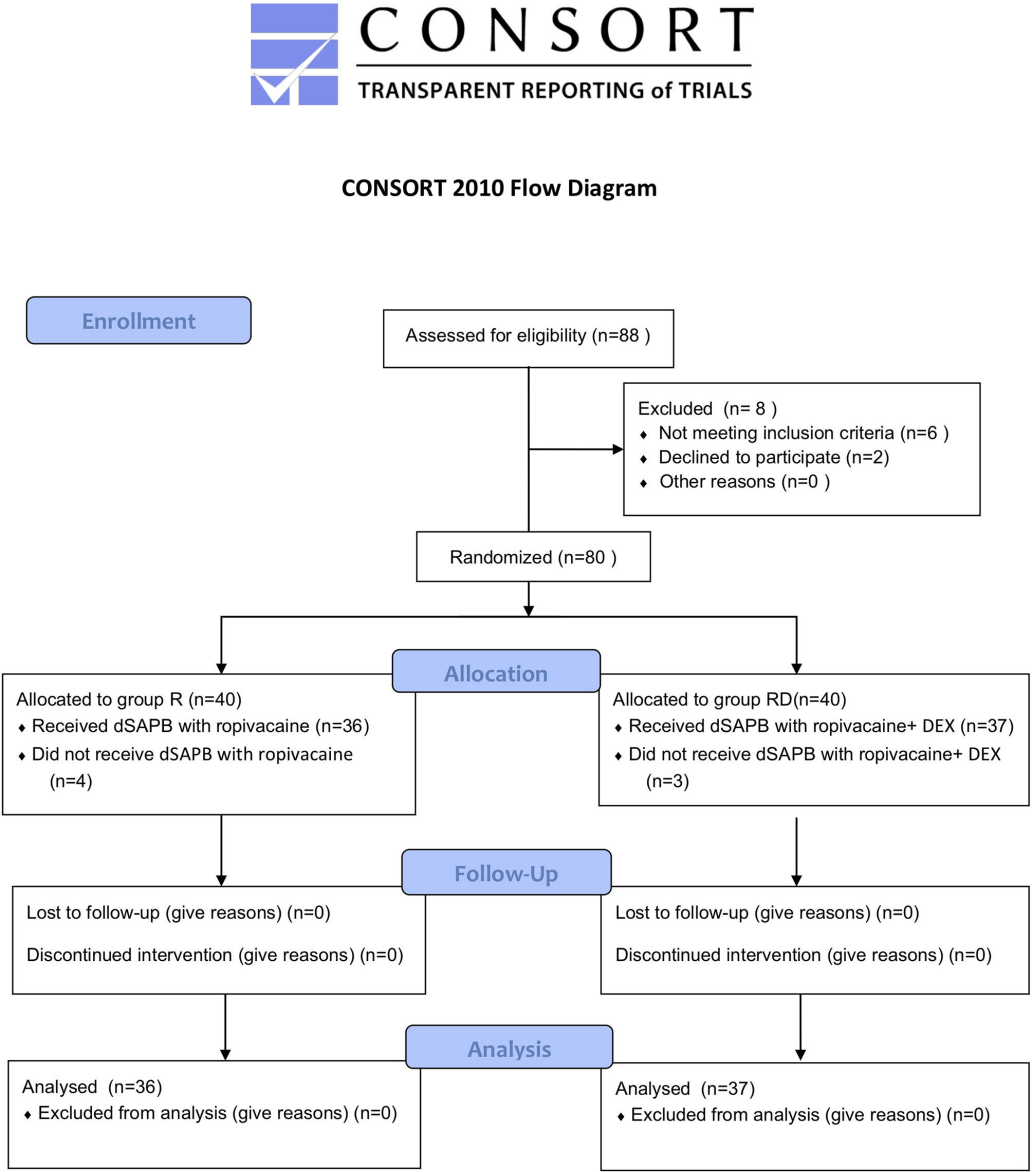
CONSORT 2010 flow diagram.

The CONSORT 2010 flowchart is shown in **Figure 1**. Between May and November 2021, we screened 88 potential participants who planned an elective unilateral modified radical mastectomy under general anesthesia. Based on inclusion criteria, six participants were deemed ineligible, and two declined to participate. A total of 80 participants participated in the trial. After randomization, four patients in the dSAPB with ropivacaine group and three in the dSAPB with DEX plus ropivacaine group were excluded for protocol violation. Therefore, data from 73 patients were used in the final analysis. Patient statistics and operation time were similar between the two groups (**Table 1**).

**Table 1:** Demographic characteristics of the participants.

Patient characteristics	Group R (n=36)	Group RD (n=37)	t or χ^2^-value	p-value
**Age (mean, year)**	54.08 (6.28)	54.62 (7.44)	−0.334	0.740
**Weight (mean, kg)**	73.97 (5.38)	72.30 (5.72)	1.288	0.202
**Height (mean, cm)**	162.69 (4.71)	162.03 (5.03)	0.585	0.560
**BMI (mean, kg/m^2^)**	27.96 (1.67)	27.57 (2.38)	0.798	0.428
**ASA**			0.134	0.892
**I**	15	16		
**II**	21	21		
**Site of surgery**			0.120	0.903
**Left**	17	18		
**Right**	19	19		
**Duration of surgery (mean, min)**	95.31 (6.22)	94.41 (5.74)	0.643	0.522
**Duration of anesthesia (mean, min)**	113.56 (7.61)	112.29 (6.94)	0.675	0.458
**Intraoperative propofol (mean, mg)**	470.27 (30.41)	464.23 (28.21)	0.882	0.382
**Intraoperative sufentanil (mean, μg)**	21.39 (3.07)	21.22 (2.98)	0.244	0.808
**Intraoperative remifentanil (mean, μg)**	151.54 (14.58)	146.74 (14.99)	1.372	0.170
**PACU stay (mean, min)**	22.06 (3.76)	22.43 (3.98)	−0.415	0.679
**Median preoperative global QoR-15 score(IQR)**	127 (123.25–131)	126 (121–130)	−0.437	0.662

**Figure 2:**
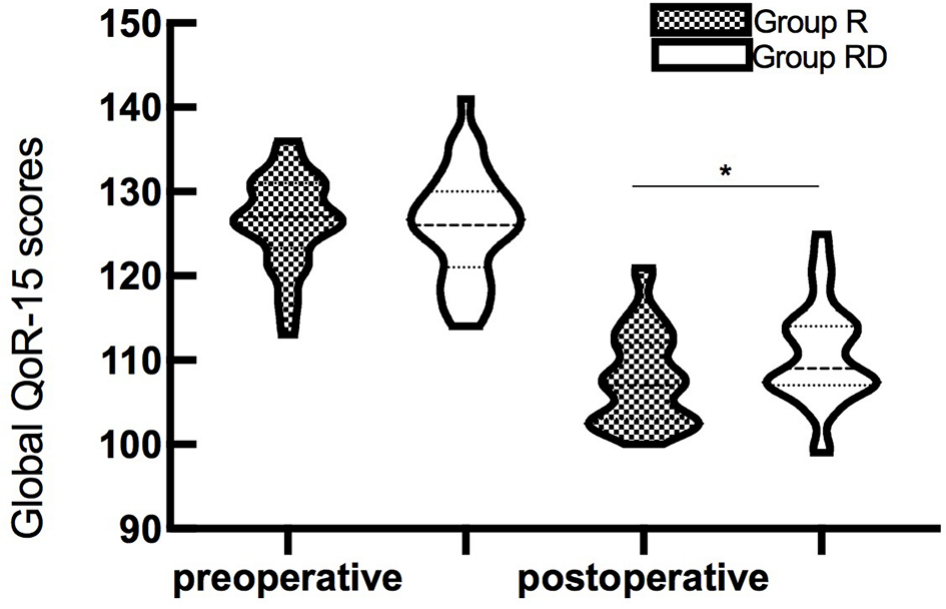
Violin plots of the global QoR-15 scores (73 patients) before surgery and 24 h after surgery. The global QoR-15 scores in the group RD were higher than those in the group R (median difference: 4, 95% CI 1–6, p=0.016 by the Mann–Whitney U-test). *p<0.05.

The global QoR-15 scores are shown in **Figure 2**. The global QoR-15 scores were significantly higher 24 h postoperatively (better recovery) in the dSAPB with ropivacaine plus DEX group than in the ropivacaine group (estimated median difference: 4; 95% CI 1–6, p=0.016).

Preoperative dSAPB administration of 0.375% ropivacaine with DEX reduced acute VAS pain scores at rest at 12th hour, exercise at 12th and 24th hour after surgery (both p < 0.05, **Table 2**). There was no significant difference between resting (p =0.125) and exercise (p =0.104) 48 h after surgery. The dSAPB of 0.375% ropivacaine with DEX group had lower cumulative consumption of opioids (sufentanil) 24 h after surgery compared with the ropivacaine group (median, 14 μg; IQR 12–15, compared with median, 14 μg; IQR 12–17; p=0.022). The median difference between the dSAPB with DEX group and the ropivacaine group was −2 μg (95% CI -2 to 0, p = 0.022).

**Table 2:** Outcomes for patients receiving deep serratus anterior plane block with ropivacaine or ropivacaine plus DEX.

	Group R (n=36)	Group RD (n=37)	z or χ^2^-value	p-value
**Primary Outcome**				
**Median postoperative global QoR-15 score(IQR)**	107 (103–112)	109.5 (107–114)	−2.414	0.016
**Secondary Outcomes**				
**VAS score in rest (Median, IQR)**				
**1th hour**	0 (0–0.5)	0 (0–0.5)	−0.624	0.533
**6th hour**	0 (0–1)	0 (0–1)	−0.109	0.914
**12th hour**	1 (1–2)	1 (1–2)	−2.136	0.033
**24th hour**	2 (2–3)	2 (2–3)	−1.909	0.056
**48th hour**	2 (1–2)	2 (1–2)	−1.534	0.125
**VAS score in movement (median, IQR)**				
**1th hour**	1 (0–1)	1 (0–1)	−0.575	0.566
**6th hour**	1 (0–1)	1 (0–1)	−1.075	0.283
**12th hour**	2 (1–3)	2 (1–3)	−2.439	0.015
**24th hour**	3 (2–3)	3 (2–3)	−2.051	0.040
**48th hour**	2 (2–3)	2 (2–3)	−1.627	0.104
**Incidence of PONV(%)**				
**24 h**	11(30.6)	6 (16.2)	2.100	0.147
**48 h**	8 (22.2)	5 (13.5)	0.945	0.331
**Patient satisfaction score (mean, SD)**	8.28 (0.70)	8.62 (0.59)	−2.254	0.024
**Bradycardia (%)**	0 (0)	1 (2.7)	0.986	0.321
**Median sufentanil rescues consumption (μg, IQR)**	14 (12–17)	14 (12–15)	−2.295	0.022
**Occurrence of dizziness(%)**	4 (11.1)	3 (8.1)	0.190	0.663
**Occurrence of delirium(%)**	0 (0)	0 (0)	NA	NA

As shown in **Table 2**, the incidence of PONV 24 and 48 h after surgery was 6 (16.2%) in the dSAPB of 0.375% ropivacaine with DEX group vs. 11 (30.6%) in the ropivacaine group, and 5 (13.5%) in the dSAPB of 0.375% ropivacaine with DEX group vs. 8(22.2%) in the ropivacaine group, and the difference was not statistically significant (p =0.147, p =0.331, respectively). Dizziness occurred in three and four patients in the dSAPB of 0.375% ropivacaine with the DEX group and the ropivacaine group, respectively.

## Discussion

Consistent with the recent focus on functional recovery, rather than simply measures of postoperative opioid consumption and pain scores (33). Our study demonstrated that the combination of dSAPB of 0.375% ropivacaine with DEX improves the QoR-15 compared with ropivacaine alone, although these improvement differences do not amount to 8, and maybe no obvious clinical significance. Therefore, we believe these differences will become more pronounced and biases will be avoided as more people are enrolled. Furthermore, participants in the dSAPB of 0.375% ropivacaine with DEX group met the superior pain relief in the early postoperative period, reducing the postoperative cumulative opioid consumption. No dSAPB -related adverse events (eg, local anesthetic toxicity, pneumothorax, bleeding or infection) were observed in the study. Taken together, these findings may still indicate that preoperative administration of dSAPB of 0.375% ropivacaine with DEX may be a useful intervention to aid recovery following breast cancer surgery.

Recovery after surgery and anesthesia is a multidimensional, inter-related, and complex process that involves many areas beyond the scope of postoperative pain (34). QoR-15 is a recently developed patient-reported outcome measure of the quality of postoperative recovery. It was developed from the QoR-40, which has been widely used and validated as a measure of the quality of postoperative recovery (35). Compared to QoR-40, QoR-15 has the same psychometric properties but is more feasible to use (28). QoR-15 is a validated quality assessment tool that can evaluate the efficacy of the post-surgical intervention on QoR-15 from a patient’s perspective (36). Meanwhile, postoperative pain remains a challenge for breast surgery patients (37); strategies to limit pain are increasingly being elaborated. Regional anesthesia technique has provided excellent postoperative analgesia and improved the quality of recovery after surgery (12). The recent emergence of ultrasound-guided regional anesthesia techniques for breast surgery includes thoracic paravertebral block, vertical ridge plane block, and thoracic nerve block, etc. provides an alternative to thoracic epidural/paraspinal block for postoperative analgesia (38). As a result, implementing patient-centered outcomes has been proposed by healthcare organizations in several countries to improve the quality of care. In this study, preoperative ultrasound-guided single-injection dSAPB of 0.375% ropivacaine plus DEX resulted in a change of 4 in QoR-15 score, indicating relevant improvement in patients’ early postoperative health status.

Dexmedetomidine has an anti-sympathetic effect that activates the vagus nerve and reduces plasma catecholamine levels, thereby providing stable hemodynamics. The analgesic effect of dexmedetomidine is the result of multiple mechanisms. A previous study suggested that the addition of dexmedetomidine to ropivacaine for sciatic nerve block in rats not only prolonged the duration of sensory and motor block of the sciatic nerve but also markedly alleviated ropivacaine−induced neurotoxicity by decreasing caspase−3−dependent sciatic nerve cell apoptosis (39). Intrathecal dexmedetomidine (5 μg) reduced the ED of intrathecal hyperbaric ropivacaine by approximately 18% for cesarean section in healthy parturients under combined spinal-epidural anesthesia (40). Lower postoperative pain scores and reduced perioperative opioid consumption are thought to be important causes. The mechanism of dexmedetomidine is mainly to activate sodium and potassium pumps to enhance membrane hyperpolarization (18). The analgesic effect of dexmedetomidine around the nerve is due to the enhancement of the cation channel activated by hyperpolarization, which prevents the neuromembrane potential from recovering from hyperpolarization to discharge in the resting state (41). Compared with placebo, dexmedetomidine prolonged sensory block time of ulnar nerve by 60% and systemic dexmedetomidine extended sensory block time by 10% (42). Therefore, local anesthetics combined with dexmedetomidine can enhance the inhibition of nerve conduction, and its analgesic effect is superior to that of local anesthetics alone.

The analgesic effect of postoperative single nerve block remains to be further elucidated due to the unsatisfactory and difficult management of indignant peripheral nerve catheter (43). Ropivacaine is a long-acting amide local anesthetic that is thought to be better at separating sensory and motor effects and less cardiotoxic (44). However, the time of using ropivacaine alone for a nerve block is short and the postoperative analgesic effect is limited. Dexmedetomidine combined with ropivacaine can enhance peripheral nerve block and prolong sensory block time (45, 46). Our present study showed that ropivacaine plus dexmedetomidine (μg/kg−1) had better analgesic efficacy than ropivacaine alone. The postoperative pain score of the RD group was significantly lower than that of the R group in the resting and exercise state, similar to previous reports of erector spinae plane block, dexmedetomidine combined with ropivacaine reduced pain scores at 24 h after breast cancer surgery (18). Our rat model (41), which showed that the combination of dexmedetomidine and ropivacaine can increase the block time of the sensory drinking motor, and the block time is related to the concentration of the dexmedetomidine and ropivacaine. On the contrary, Abdallah et al. suggested that the addition of deep serratus anterior block to general analgesia cannot improve the quality of rehabilitation in patients undergoing outpatient breast cancer surgery (47), this may be related to the small number of cases enrolled and the selection of patients with larger weight (BMI < 35) undergoing ambulatory surgery. Patients with antepartum obesity may require decreased ropivacaine concentration for epidural labor analgesia when co-administered with 0.5 µg/ml dexmedetomidine (48). However, it was a very meaningful study, and more studies are needed to confirm the effect of serratus anterior block in breast cancer surgery.

Reducing perioperative opioid use is one of the current goals of enhanced recovery after surgery, which aims to reduce potential opioid-related side effects. Regional nerve block plays an important role in opioid reduction. In this study, dexmedetomidine combined with ropivacaine can reduce the postoperative dosage of sufentanil and improve patient satisfaction. Previous reports indicate that dexmedetomidine combined with a paravertebral block was found to reduce opioid dosage during thoracoscopic surgery (49). Paraspinal thoracic block can reduce the consumption of opioids in early postoperative breast surgery, which may be related to the anesthetic time of ropivacaine (50) avoided the complications of epidural block in breast surgery patients (51). Unilateral ESPB was administered during modified radical mastectomy, similar to a unilateral thoracic epidural block and without hemodynamic side effects, can reduce the use of opioids after breast cancer (52).

### Limitations

First, despite the results showing a statistically significant difference in postoperative QoR-15 scores between the two groups, the difference was not clinically significant. More patient enrollment is needed to reduce this bias. Second, this study is a single-center randomized controlled study, which requires more centers and more cases to verify our conclusions. Third, we did not investigate the different doses of dexmedetomidine on the intrathecal ropivacaine for modified radical mastectomy. Fourth, the study also did not evaluate preoperative skin diffusion of the blocker as a proxy for analgesic efficacy. Fifth, we did not regulate the postoperative pain management of patients after discharge from the hospital. With focusing on short-term pain, we also need to focus on chronic pain in patients. The last but not least, some confounding factors, such as environment, anxiety, stress, and post-traumatic stress disorder, may affect the resolution of postoperative pain. We did not include all confounding factors affecting postoperative pain, and further relevant studies are needed.

## Conclusions

In conclusion, under the conditions of the present study, the ultrasound-guided deep serratus anterior plane block combined with dexmedetomidine plus ropivacaine may improve the QoR-15 with ropivacaine alone in patients undergoing modified radical mastectomy and indicate that it may be a useful intervention to aid recovery following breast cancer surgery. Furthermore, participants in the ropivacaine with DEX group met the superior pain relief in the early postoperative period, reduced the postoperative cumulative opioid consumption, and increased patient satisfaction, and there was no increase in the incidence of complications.

## Data Availability Statement

The raw data supporting the conclusions of this article will be made available by the authors, without undue reservation.

## Ethics Statement

The studies involving human participants were reviewed and approved by the ethics committee of Bethune International Peace Hospital. The patients/participants provided their written informed consent to participate in this study.

## Author Contributions

YW was involved in the conception and design of the study. YK collected the data. YL prepared the placebo tablets. BF analyzed the data. YW drafted the first and final version of the manuscript. All authors contributed to the article and approved the submitted version.

## Conflict of Interest

The authors declare that the research was conducted in the absence of any commercial or financial relationships that could be construed as a potential conflict of interest.

## Publisher’s Note

All claims expressed in this article are solely those of the authors and do not necessarily represent those of their affiliated organizations, or those of the publisher, the editors and the reviewers. Any product that may be evaluated in this article, or claim that may be made by its manufacturer, is not guaranteed or endorsed by the publisher.

## References

[1] Siegel RL Miller KD Fuchs HE Jemal A. Cancer Statistics, 2021. CA Cancer J Clin (2021) 71(1):7–33. doi: 10.3322/caac.21654 33433946

[2] Organization WH. Latest Global Cancer Data: Cancer Burden Rises to 19.3 Million New Cases and 10.0 Million Cancer Deaths in 2020 (2020). Available at: https://iarc.who.int/wp-content/uploads/2020/12/pr292_E.pdf.

[3] Barzaman K Karami J Zarei Z Hosseinzadeh A Kazemi MH Moradi-Kalbolandi S. Breast Cancer: Biology, Biomarkers, and Treatments. Int Immunopharmacol (2020) 84:106535. doi: 10.1016/j.intimp.2020.106535 32361569

[4] Duggan C Trapani D Ilbawi AM Fidarova E Laversanne M Curigliano G. National Health System Characteristics, Breast Cancer Stage at Diagnosis, and Breast Cancer Mortality: A Population-Based Analysis. Lancet Oncol (2021) 22(11):1632–42. doi: 10.1016/S1470-2045(21)00462-9 34653370

[5] Sung H Ferlay J Siegel RL Laversanne M Soerjomataram I Jemal A. Global Cancer Statistics 2020: GLOBOCAN Estimates of Incidence and Mortality Worldwide for 36 Cancers in 185 Countries. CA Cancer J Clin (2021) 71(3):209–49. doi: 10.3322/caac.21660 33538338

[6] Nounou MI ElAmrawy F Ahmed N Abdelraouf K Goda S Syed-Sha-Qhattal H. Breast Cancer: Conventional Diagnosis and Treatment Modalities and Recent Patents and Technologies. Breast Cancer (Auckl) (2015) 9(Suppl 2):17–34. doi: 10.4137/BCBCR.S29420 26462242 PMC4589089

[7] Yan J Liu Z Du S Li J Ma L Li L. Diagnosis and Treatment of Breast Cancer in the Precision Medicine Era. Methods Mol Biol (2020) 2204 p:53–61. doi: 10.1007/978-1-0716-0904-0_5 32710314

[8] Benjamin DJ. The Efficacy of Surgical Treatment of Cancer - 20 Years Later. Med Hypotheses (2014) 82(4):412–20. doi: 10.1016/j.mehy.2014.01.004 24480434

[9] Lopez M Padilla ML Garcia B Orozco J Rodilla AM. Prevention of Acute Postoperative Pain in Breast Cancer: A Comparison Between Opioids Versus Ketamine in the Intraoperatory Analgesia. Pain Res Manag (2021) 2021:3290289. doi: 10.1155/2021/3290289 34840635 PMC8612786

[10] Cata JP Corrales G Speer B Owusu-Agyemang P. Postoperative Acute Pain Challenges in Patients With Cancer. Best Pract Res Clin Anaesthesiol (2019) 33(3):361–71. doi: 10.1016/j.bpa.2019.07.018 31785721

[11] Aneurin Moorthy ANE Donal J. Buggy, Can Acute Postoperative Pain Management After Tumour Resection Surgery Modulate Risk of Later Recurrence or Metastasis? Front Oncol (2021). doi: 10.3389/fonc.2021.802592 PMC8716859 34976840

[12] Woodworth GE Ivie RMJ Nelson SM Walker CM Maniker RB. Perioperative Breast Analgesia: A Qualitative Review of Anatomy and Regional Techniques. Reg Anesth Pain Med (2017) 42(5):609–31. doi: 10.1097/AAP.0000000000000641 28820803

[13] Clary Z Nazir N Butterworth J. Transversus Abdominis Plane Block With Liposomal Bupivacaine Versus Thoracic Epidural for Postoperative Analgesia After Deep Inferior Epigastric Artery Perforator Flap-Based Breast Reconstruction. Ann Plast Surg (2020) 85(6):e24–6. doi: 10.1097/SAP.0000000000002423 33170580

[14] Arai YC Nishihara M Aono S Ikemoto T Suzuki C Kinoshita A. Pulsed Radiofrequency Treatment Within Brachial Plexus for the Management of Intractable Neoplastic Plexopathic Pain. J Anesth (2013) 27(2):298–301. doi: 10.1007/s00540-012-1501-8 23070568

[15] Offodile AC 2nd Sheckter CC Tucker A Watzker A Ottino K Zammert M. Preoperative Paravertebral Blocks for the Management of Acute Pain Following Mastectomy: A Cost-Effectiveness Analysis. Breast Cancer Res Treat (2017) 165(3):477–84. doi: 10.1007/s10549-017-4371-9 28677010

[16] Thomas M Philip FA Mathew AP Jagathnath Krishna KM. Intraoperative Pectoral Nerve Block (Pec) for Breast Cancer Surgery: A Randomized Controlled Trial. J Anaesthesiol Clin Pharmacol (2018) 34(3):318–23. doi: 10.4103/joacp.JOACP_191_17 PMC6194828 30386013

[17] Battista C Krishnan S. Pectoralis Nerve Block. Treasure Island (FL: StatPearls (2021). 31613471

[18] Wang X Ran G Chen X Xie C Wang J Liu X. The Effect of Ultrasound-Guided Erector Spinae Plane Block Combined With Dexmedetomidine on Postoperative Analgesia in Patients Undergoing Modified Radical Mastectomy: A Randomized Controlled Trial. Pain Ther (2021) 10(1):475–84. doi: 10.1007/s40122-020-00234-9 PMC8119550 33475952

[19] Blanco R Parras T McDonnell JG Prats-Galino A. Serratus Plane Block: A Novel Ultrasound-Guided Thoracic Wall Nerve Block. Anaesthesia (2013) 68(11):1107–13. doi: 10.1111/anae.12344 23923989

[20] Xie C Ran G Chen D Lu Y. A Narrative Review of Ultrasound-Guided Serratus Anterior Plane Block. Ann Palliat Med (2021) 10(1):700–6. doi: 10.21037/apm-20-1542 33440981

[21] Xiao YK She SZ Xu LX Zheng B. Serratus Anterior Plane Block Combined With General Analgesia and Patient-Controlled Serratus Anterior Plane Block in Patients With Breast Cancer: A Randomized Control Trial. Adv Ther (2021) 38(6):3444–54. doi: 10.1007/s12325-021-01782-y 34021888

[22] Arora S Ovung R Bharti N Yaddanapudi S Singh G. Efficacy of Serratus Anterior Plane Block Versus Thoracic Paravertebral Block for Postoperative Analgesia After Breast Cancer Surgery - A Randomized Trial. Braz J Anesthesiol (2021) S0104-14(21):00376. doi: 10.1016/j.bjane.2021.09.017 PMC9515677 34627832

[23] Kunigo T Murouchi T Yamamoto S Yamakage M. Injection Volume and Anesthetic Effect in Serratus Plane Block. Reg Anesth Pain Med (2017) 42(6):737–40. doi: 10.1097/AAP.0000000000000649 28891826

[24] Edwards JT Langridge XT Cheng GS McBroom MM Minhajuddin A Machi AT. Superficial vs. Deep Serratus Anterior Plane Block for Analgesia in Patients Undergoing Mastectomy: A Randomized Prospective Trial. J Clin Anesth (2021) 75:110470. doi: 10.1016/j.jclinane.2021.110470 34364099

[25] Mahmoud M Mason KP. Dexmedetomidine: Review, Update, and Future Considerations of Paediatric Perioperative and Periprocedural Applications and Limitations. Br J Anaesth (2015) 115(2):171–82. doi: 10.1093/bja/aev226 26170346

[26] Vorobeichik L Brull R Abdallah FW. Evidence Basis for Using Perineural Dexmedetomidine to Enhance the Quality of Brachial Plexus Nerve Blocks: A Systematic Review and Meta-Analysis of Randomized Controlled Trials. Br J Anaesth (2017) 118(2):167–81. doi: 10.1093/bja/aew411 28100520

[27] Hussain N Grzywacz VP Ferreri CA Atrey A Banfield L Shaparin N. Investigating the Efficacy of Dexmedetomidine as an Adjuvant to Local Anesthesia in Brachial Plexus Block: A Systematic Review and Meta-Analysis of 18 Randomized Controlled Trials. Reg Anesth Pain Med (2017) 42(2):184–96. doi: 10.1097/AAP.0000000000000564 28178091

[28] Stark PA Myles PS Burke JA. Development and Psychometric Evaluation of a Postoperative Quality of Recovery Score: The QoR-15. Anesthesiology (2013) 118(6):1332–40. doi: 10.1097/ALN.0b013e318289b84b 23411725

[29] Barrington MJ Seah GJ Gotmaker R Lim D Byrne K. Quality of Recovery After Breast Surgery: A Multicenter Randomized Clinical Trial Comparing Pectoral Nerves Interfascial Plane (Pectoral Nerves II) Block With Surgical Infiltration. Anesth Analg (2020) 130(6):1559–67. doi: 10.1213/ANE.0000000000004371 31490251

[30] Aldrete JA. The Post-Anesthesia Recovery Score Revisited. J Clin Anesth (1995) 7(1):89–91. doi: 10.1016/0952-8180(94)00001-K 7772368

[31] Farooq F Khan R Ahmed A. Assessment of Patient Satisfaction With Acute Pain Management Service: Monitoring Quality of Care in Clinical Setting. Indian J Anaesth (2016) 60(4):248–52. doi: 10.4103/0019-5049.179450 PMC4840804 27141107

[32] Myles PS Myles DB Galagher W Chew C MacDonald N Dennis A. Minimal Clinically Important Difference for Three Quality of Recovery Scales. Anesthesiology (2016) 125(1):39–45. doi: 10.1097/ALN.0000000000001158 27159009

[33] Sites BD. Editor’s Note: ‘Getting Closer to the Truth-Together’. Reg Anesth Pain Med (2019) 44(11):979–80. doi: 10.1136/rapm-2019-100928 31624221

[34] Nair G Wong DJ Chan E Alexander T Jeevananthan R Pawa A. Mode of Anesthesia and Quality of Recovery After Breast Surgery: A Case Series of 100 Patients. Cureus (2021) 13(3):e13822. doi: 10.7759/cureus.13822 33859887 PMC8038898

[35] Gornall BF Myles PS Smith CL Burke JA Leslie K Pereira MJ. Measurement of Quality of Recovery Using the QoR-40: A Quantitative Systematic Review. Br J Anaesth (2013) 111(2):161–9. doi: 10.1093/bja/aet014 23471753

[36] Myles PS Myles DB. An Updated Minimal Clinically Important Difference for the QoR-15 Scale. Anesthesiology (2021) 135(5):934–5. doi: 10.1097/ALN.0000000000003977 34543410

[37] Rajput K Reid CB Yanez D Shiwlochan D Ohanyan S Chow R. Patterns of Use of Opioid Sparing Adjuncts for Perioperative Pain Management of Patients on Chronic Opioids. Pain Physician (2021) 24(8):577–86. 34793645

[38] Thiruvenkatarajan V Cruz Eng H Adhikary SD. An Update on Regional Analgesia for Rib Fractures. Curr Opin Anaesthesiol (2018) 31(5):601–7. doi: 10.1097/ACO.0000000000000637 30020155

[39] Xue X Fan J Ma X Liu Y Han X Leng Y. Effects of Local Dexmedetomidine Administration on the Neurotoxicity of Ropivacaine for Sciatic Nerve Block in Rats. Mol Med Rep (2020) 22(5):4360–6. doi: 10.3892/mmr.2020.11514 PMC7533505 33000208

[40] Tang Y Yang M Fu F Huang X Feng Y Chen X. Comparison of the ED50 of Intrathecal Hyperbaric Ropivacaine Co-Administered With or Without Intrathecal Dexmedetomidine for Cesarean Section: A Prospective, Double-Blinded, Randomized Dose-Response Trial Using Up-Down Sequential Allocation Method. J Clin Anesth (2020) 62:109725. doi: 10.1016/j.jclinane.2020.109725 32036258

[41] Brummett CM Padda AK Amodeo FS Welch KB Lydic R. Perineural Dexmedetomidine Added to Ropivacaine Causes a Dose-Dependent Increase in the Duration of Thermal Antinociception in Sciatic Nerve Block in Rat. Anesthesiology (2009) 111(5):1111–9. doi: 10.1097/ALN.0b013e3181bbcc26 PMC2770892 19858875

[42] Marhofer D Kettner SC Marhofer P Pils S Weber M Zeitlinger M. Dexmedetomidine as an Adjuvant to Ropivacaine Prolongs Peripheral Nerve Block: A Volunteer Study. Br J Anaesth (2013) 110(3):438–42. doi: 10.1093/bja/aes400 23161360

[43] Koraki E Stachtari C Kapsokalyvas I Stergiouda Z Katsanevaki A Trikoupi A. Dexmedetomidine as an Adjuvant to 0.5% Ropivacaine in Ultrasound-Guided Axillary Brachial Plexus Block. J Clin Pharm Ther (2018) 43(3):348–52. doi: 10.1111/jcpt.12657 29193234

[44] George AM Liu M. Ropivacaine. Treasure Island (FL: StatPearls (2021).

[45] Qian M Gao F Liu J Xu P. Dexmedetomidine Versus Fentanyl as Adjuvants to Ropivacaine for Epidural Anaesthesia: A Systematic Review and Meta-Analysis. Int J Clin Pract (2021) 75(5):e13772. doi: 10.1111/ijcp.13772 33078536

[46] Rao J Gao Z Qiu G Gao P Wang Q Zhong W. Nalbuphine and Dexmedetomidine as Adjuvants to Ropivacaine in Ultrasound-Guided Erector Spinae Plane Block for Video-Assisted Thoracoscopic Lobectomy Surgery: A Randomized, Double-Blind, Placebo-Controlled Trial. Med (Baltimore) (2021) 100(32):e26962. doi: 10.1097/MD.0000000000026962 PMC8360433 34397949

[47] Abdallah FW Patel V Madjdpour C Cil T Brull R. Quality of Recovery Scores in Deep Serratus Anterior Plane Block vs. Sham Block in Ambulatory Breast Cancer Surgery: A Randomised Controlled Trial. Anaesthesia (2021) 76(9):1190–7. doi: 10.1111/anae.15373 33492696

[48] Chen X Cai M Lei X Yu J. Obesity Decreases the EC50 of Epidural Ropivacaine When Combined With Dexmedetomidine for Labor Analgesia. Expert Rev Clin Pharmacol (2021) 14(8):1051–6. doi: 10.1080/17512433.2021.1929924 33980116

[49] Hong B Lim C Kang H Eom H Kim Y Cho HJ. Thoracic Paravertebral Block With Adjuvant Dexmedetomidine in Video-Assisted Thoracoscopic Surgery: A Randomized, Double-Blind Study. J Clin Med (2019) 8(3):352. doi: 10.3390/jcm8030352 PMC6462904 30871093

[50] Piccioni F Segat M Falini S Umari M Putina O Cavaliere L. Enhanced Recovery Pathways in Thoracic Surgery From Italian VATS Group: Perioperative Analgesia Protocols. J Thorac Dis (2018) 10(Suppl 4):S555–63. doi: 10.21037/jtd.2017.12.86 PMC5880986 29629202

[51] Almeida CR. Parascapular Sub-Iliocostalis Plane Block: Comparative Description of a Novel Technique for Posterior Rib Fractures. Pain Pract (2021) 21(6):708–14. doi: 10.1111/papr.13003 33586285

[52] Thiagarajan P Thota RS Divatia JV. Efficacy of Ultrasound-Guided Erector Spinae Plane Block Following Breast Surgery - A Double-Blinded Randomised, Controlled Study. Indian J Anaesth (2021) 65(5):377–82. doi: 10.4103/ija.IJA_1426_20 PMC8202792 34211195

